# Selection Pressure in CD8^+^ T-cell Epitopes in the *pol* Gene of HIV-1 Infected Individuals in Colombia. A Bioinformatic Approach

**DOI:** 10.3390/v7031313

**Published:** 2015-03-20

**Authors:** Liliana Acevedo-Sáenz, Rodrigo Ochoa, Maria Teresa Rugeles, Patricia Olaya-García, Paula Andrea Velilla-Hernández, Francisco J. Diaz

**Affiliations:** 1 Grupo Inmunovirología, Facultad de Medicina, Universidad de Antioquia UdeA, Calle 70 No. 52-21, Medellín, 050010, Colombia; E-Mails: liloacevedo@gmail.com (L.A.-S.); maria.rugeles@udea.edu.co (M.T.R.); pvelilla@gmail.com (P.A.V.-H.); 2 Programa de Estudio y Control de Enfermedades Tropicales—PECET, Universidad de Antioquia, Medellín, 050010, Colombia; E-Mail: rodrigo.ochoa@udea.edu.co; 3 Centro de Análisis Molecular, Bogotá D.C., 111156, Colombia; E-Mail: patolaya@etb.net.co

**Keywords:** human immunodeficiency virus, positive selection, epitopes, docking, CD8^+^ T-cells

## Abstract

One of the main characteristics of the human immunodeficiency virus is its genetic variability and rapid adaptation to changing environmental conditions. This variability, resulting from the lack of proofreading activity of the viral reverse transcriptase, generates mutations that could be fixed either by random genetic drift or by positive selection. Among the forces driving positive selection are antiretroviral therapy and CD8^+^ T-cells, the most important immune mechanism involved in viral control. Here, we describe mutations induced by these selective forces acting on the *pol* gene of HIV in a group of infected individuals. We used Maximum Likelihood analyses of the ratio of non-synonymous to synonymous mutations per site (d_N_/d_S_) to study the extent of positive selection in the protease and the reverse transcriptase, using 614 viral sequences from Colombian patients. We also performed computational approaches, docking and algorithmic analyses, to assess whether the positively selected mutations affected binding to the HLA molecules. We found 19 positively-selected codons in drug resistance-associated sites and 22 located within CD8^+^ T-cell epitopes. A high percentage of mutations in these epitopes has not been previously reported. According to the docking analyses only one of those mutations affected HLA binding. However, algorithmic methods predicted a decrease in the affinity for the HLA molecule in seven mutated peptides. The bioinformatics strategies described here are useful to identify putative positively selected mutations associated with immune escape but should be complemented with an experimental approach to define the impact of these mutations on the functional profile of the CD8^+^ T-cells.

## 1. Introduction

Human immunodeficiency virus 1 (HIV-1) is characterized by high levels of diversity and rapid evolution. For HIV-1, four genetic groups have been described: group M causes most of the infections worldwide [1] and is divided into nine subtypes: A–D, F–H, J and K [2]; groups O, N and P which are restricted to the West and Central Africa and have a lower epidemiological impact [3,4,5]. Intersubtype recombinants originated in individuals co-infected with two or more subtypes are also common [6]. Two studies carried out in the cities of Medellín and Bogotá (Colombia) with HIV sequences of 2001 and 2002 classified the circulating strains within subtype B [7,8].

The genetic diversity of HIV is partially due to the high rates of replication and mutation [9]. The lack of proofreading activity of the viral reverse transcriptase (RT) is the major mechanism of mutation [10,11]. The mutation rate is defined as the number of substitutions per nucleotide site per replicative cycle (s/s/c); in HIV, this rate varies between 2.4 × 10^−5^ and 3.4 × 10^−5^ (s/s/c) [12,13,14]. From this, it follows that between one third and one fourth of the new viral particles carry at least one mutation.

These mutations contribute to HIV diversification and have been associated with evasion of the immune response [15], defective virus particles [16,17], changes of cellular tropism and resistance to antiretroviral drugs [18]. Some mutations are positively selected by the antiretroviral therapy or the immune response, driving HIV evolution [19,20]. Specifically, CD8^+^ T-cell responses to HIV-1, restricted through human leukocyte antigen (HLA) molecules, are responsible for the positive selection of escape mutations observed in HIV [21,22]. These mutations are often located in sites of inter- and intra-subtype variability [23]. The rapid generation of escape mutants by HIV-1 strains is an enormous challenge to be overcome by the immune system, and also in the design of a globally effective HIV vaccine [24].

Moore and collaborators determined the association between specific HLA alleles in HIV-1-infected individuals and the presence or absence of some amino acid replacements in the virus sequences at particular positions [22]; this study confirmed selection of escape mutations restricted by HLA class I molecules, and underscores the high proportion of viral codons whose evolution is driven by immune escape. In fact, between 10% and 40% of the variation in codons of HIV-1 subtype B strains is driven by immune pressure associated with class I HLA alleles [25]. The clinical implication of these mutations associated with the immune escape is the increase in the rate of progression of the infection; this phenomenon has most frequently been observed in individuals expressing HLA-B*27 and HLA-B*51 in which amino acid substitutions in immunodominant epitopes led to high levels of HIV replication and rapid progression to AIDS [26,27,28].

The aim of our study was to explore the presence of mutations presumably associated with immune escape in the protease (PRO) and RT proteins of HIV-1 of chronically infected individuals in Colombia. For this purpose, we designed an *in silico* strategy consisting in detecting positively selected mutations in T CD8^+^ epitopes, followed by the prediction of changes in the affinity between the mutated peptide and the corresponding HLA molecule. We identified some selected peptides not previously reported as escape mutants within epitopes restricted to class I HLA molecules with high prevalence in the Colombian population and predicted their consequences in the process of antigenic presentation.

## 2. Materials and Methods

### 2.1. Data Sources and Sequence Alignments

We analyzed 614 sequences from the *pol* gene (genome positions 2262–2549 and 2661–3290) coding for the PRO and part of the RT. These sequences were provided by the Centro de Análisis Molecular, a specialized laboratory located in Bogotá, the capital, which performed antiviral sensitivity tests in samples from different regions of Colombia. These samples were collected between 2000 and 2007 from individuals receiving antiretroviral therapy. Sequencing was performed by the TRUGENE^®^ kit (Siemens Healthcare Diagnostics, Tarrytown, NY, USA) [29]. A code was assigned to each sequence in order to be used anonymously; clinical data and result of additional laboratory studies were not available. Sequences were aligned by the Clustal W method implemented in the BioEdit package (Ibis Bioscience, Carlsbad, CA, USA), using the reference sequence HXB2 of HIV-1 (GenBank accession number K03455.1). Analysis of antiviral resistance data, subtyping and phylodynamics of this database have already been published [30,31]. Most (82%) sequences exhibit resistance to at least one antiviral drug [30]. Almost all of these *pol* gene sequences were subtype B; the exception was one subtype F. No evidence of inter-subtype recombination was found [31].

### 2.2. Tests of Positive Selection

We first used the *Z*-test, as implemented in the MEGA 5.2.2 software [32], to explore evidence of natural selection in the dataset. The statistics (d_N_–d_S_), where d_S_ and d_N_ are the numbers of synonymous substitutions per synonymous site and nonsynonymous substitutions per nonsynonymous site, respectively, were estimated using the Nei-Gojobori method [33]. The variance of the difference was computed using the bootstrap method (10,000 replicates).

We also searched for natural selection by HyPhy, a Maximum Likelihood (ML) based method [34]. For each codon, estimates of d_S_ and d_N_ were produced using the joint ML reconstructions of ancestral states under the Muse-Gaut model [35] of codon substitution and the General Time Reversible model [36] of nucleotide substitution. Positive selection was inferred for codons with d_N_/d_S_ values significantly greater than 1 and the probability of rejecting the null hypothesis of neutral evolution (*p*-value) was calculated [37,38]; *p* values < 0.05 were considered significant.

In both the *Z*-test and HyPhy analyses, all positions with less than 90% site coverage were eliminated. That is, fewer than 10% alignment gaps, missing data, and ambiguous bases were allowed at any position. There was a total of 304 codons in the final dataset.

To confirm the results obtained with the MEGA implementation of HyPhy, the Single Likelihood Ancestor Counting (SLAC) method implemented in the Datamonkey webserver [37] was used [39]. Since Datamonkey does not process datasets with more than 500 sequences, for this analysis the sequences were arbitrarily divided in two groups. The nucleotide and codons substitution models were the same used in the analysis performed in MEGA 5.2.2 software.

### 2.3. Identification of Peptides

We identified HLA-class I restricted epitopes using the HIV Molecular Immunology Database of Los Alamos [40]. Each epitope was located in the reference sequence HXB2 of HIV-1 (GenBank accession number: K03455.1) and compared to the homologous sequences in our dataset. For each epitope sequence, we identified the mutations, measured their frequency and classified them as susceptible forms (SF), immune escape (IE) or drug resistance associated (DRAS) mutations. Susceptible forms, defined as epitopes with mutations that are still able to induce a CD8^+^ T-cell response, and immune escape, defined as mutated epitopes with documented or inferred escape capabilities, were derived from the Los Alamos Database. Substitutions were considered as drug resistance mutations if they were included in the Stanford HIV drug resistance database [41,42] or in the Update of the Drug Resistance Mutations in HIV-1 of the International AIDS Society [43]; other substitutions observed but not included in any of the previous definitions were dubbed as “non-classified” (NC) mutations.

### 2.4. Collection and Preparation of the Three-Dimensional Structure of HLA Molecules

From the Protein Data Bank (PDB) database [44] we downloaded the three-dimensional structure of certain HLA molecules found with high frequency in Colombia. We only considered proteins with a resolution ≤ 2.0 Å. The proteins were edited using the AutoDock Tools program [45]; external ligands and crystallized water were eliminated. AutoDock tools were used to add hydrogen to polar atoms of the molecules, and Gasteiger changes to HLA proteins, facilitating its potential interactions with the peptides. The PDB codes were 1E27, 1DUZ, 3UTQ, 3MRK, 1EFX, 1M6O, 3BO8, 3RL1, 1XR9, 3C9N and 4HX1.

### 2.5. Structural Prediction of Peptides

Since we required the 3D structures of all peptides included in the study for the *in silico* docking simulation assay, we used the homology-modeling I-TASSER [46,47] and ESyPred3D [48] servers. With this strategy, we predicted the 3D structure of the wild type (WT) and mutated peptides in order to assess how the mutations affect their folding and the affinity of their binding to the MHC molecules.

### 2.6. HLA-Peptide Binding Predictions

We predicted the affinities between the HLA protein and the WT or mutant peptides using the receptor-ligand docking simulation AutoDockVina software [49] and three different algorithms of affinity estimation: NetMHCpan 2.8 [50,51], NetMHC [52] and stabilized matrix method (SMM) [53,54]. For AutoDockVina, we evaluated the affinity constant based on the indirect score of the free energy of binding. The conformational search space in the AutoDockVina simulation was limited to the vicinity of the major histocompatibility complex (MHC) pocket. For each peptide, the docking simulations were run 20 times and the best scores of each run were selected and averaged; the interactions between residues from both, ligand and receptor were verified in detail. Statistical comparison of the free energy of binding of the WT and mutated epitopes were performed by the Kruskal-Wallis and Mann-Whitney U tests.

For the NetMHCpan, NetMHC and SMM algorithms, we considered as good, intermediate and poor bindings when the Inhibitory Concentration 50 (IC_50_) was < 50 nM, 50–500 nM or > 500 nM, respectively. Two-fold or larger differences between the affinities of the WT and mutated peptides with the corresponding HLA molecule were considered as significant [55]. Correlation analyses among the different methods of affinity assessment were performed by the Spearman test. All statistics were performed using GraphPrism 5.0 package; *p*-values lower than 0.05 were considered significant.

## 3. Results

### 3.1. Z-Test and Maximum Likelihood Analysis of Positive Selection

We analyzed 614 *pol* gene sequences corresponding to the PRO (codons 4–99) and the RT (codons 38–247) obtained from HIV-infected individuals living in 16 Colombian cities, mainly Bogotá (44.4%), Cali (12.6%) and Medellín (10.6%). There were 280 variable sites out of a total of 304 codons.

Initially, we searched for evidence of selection using the *Z*-test in the overall average of all sequences. The null hypothesis of strict neutrality (d_N_ = d_S_) was rejected in favor of the alternative hypothesis (d_N_ ≠ d_S_) with a *p* = 0.000. The value for the statistic d_N_–d_S_ was −10.5, indicating a higher rate of synonymous than non-synonymous substitutions. When tested for positive selection the null hypothesis of neutrality in favor of the alternative (d_N_ > d_S_) could not be rejected (*p* = 1.000). Conversely, when tested for purifying selection the null hypothesis of neutrality was rejected (*p* = 0.000) in favor of the alternative (d_N_ < d_S_), indicating that negative selection predominates along the studied segments of *pol*.

However, it is well known that a purifying selection in most parts of the sequence could hide positive selection occurring at specific codons [33,36]. To obtain an assessment of codon-specific selective pressures, we performed an analysis of d_N_/d_S_ by the Maximum Likelihood method using the HyPhy implementation in MEGA. The test of positive selection was significant (*p* < 0.05) in 35 codons (18 in PRO and 17 in RT) (Table 1). For both proteins, sites under positive selection were spread throughout the gene.

**Table 1:** Mutations identified as positively selected by the Maximum Likelihood method in protease (PRO) and reverse transcriptase (RT) of 614 human immunodeficiency virus (HIV) infected individuals.

Codons Position (HXB2)	Substitution	d_N_/d_S_^a^	*p* Value	Location ^b^
Protease				
12	T → P/S	4.4	0.0000 *	Epitope
13	I → V	5.0	0.0002	Epitope
19	L → I/Q/V	1.7	0.0015	Epitope
35	E → D	1.9	0.0038	Epitope
37	S→N/E/D	5.7	0.0000 *	Epitope
41	R→K	2.9	0.0000 *	Epitope
54	I → L/V/M/T/A/S	3.0	0.0000 *	Epitope/DRAS
62	I → V	2.9	0.0002	DRAS
64	I → L/M/V	6.8	0.0000 *	DRAS
71	A → V/I/T/L	2.3	0.0000 *	DRAS
72	I → V/T/L/R	2.1	0.0115	Epitope
73	G → C/S/T/A	2.1	0.0146	DRAS
74	T → P	15.6	0.0000 *	DRAS
77	V → I	5.3	0.0000 *	Epitope/DRAS
82	V → A/F/S/T	4.2	0.0000 *	Epitope/DRAS
85	I → V	36	0.0460	DRAS
90	L → M	4.4	0.0000 *	DRAS
93	I → L/M	8.2	0.0000 *	DRAS
Reverse transcriptase				
39	T → A/K/S/L	4.4	0.0000 *	Epitope
48	S → T	2.6	0.0203	Epitope
69	T → S/N/D/A/G	2.2	0.0008	DRAS
74	L → V/I	3.0	0.0000 *	Epitope/DRAS
75	V → I	2.4	0.0024	DRAS
98	A → S	2.0	0.0106	Epitope
102	K → R/Q/E/N/H	20.1	0.0000 *	DRAS
103	K → N/S	2.0	0.0003	DRAS
118	V → I	1.7	0.0179	Epitope
135	I → T/V/L/R/M/K	3.3	0.0000 *	Epitope
162	S → C/A/Y/D/N/H	2.1	0.0003	Epitope
184	M → V	2.5	0.0000 *	Epitope/DRAS
188	Y → L	4.8	0.0002	DRAS
200	T → A/I/E	17.4	0.0000 *	Epitope
202	I → V	606.1	0.0083	Epitope
211	R → K/Q/G/T	1.5	0.0051	Epitope
215	T → I	3.4	0.0000 *	Epitope

^a^ Ratio of nonsynonymous (d_N_) to synonymous (d_S_) substitutions per site. ^b^ DRAS: Drug resistance associated sites according to the Stanford HIV drug resistance database [41,42] or the last Update of the Drug Resistance Mutations in HIV-1 of the International AIDS Society [43]. Epitope: peptide recognized by CD8^+^ T-cells according to the HIV Molecular Immunology Database of Los Alamos [40]. * *p*-values below the Bonferroni corrected (0.00016) significance level.

Eleven out of 18 selected codons in the PRO and 7 out of 17 in the RT have been previously associated with drug resistance; one of them (position 102 in the RT) was associated with drug resistance in a previous report, although it is not included in the last update of the Drug Resistance Mutations in HIV-1 or in the Stanford HIV drug resistance database [43,56,57]. On the other hand, 10 of the selected codons in the PRO and 12 in the RT were located in recognized CD8^+^ T-cell epitopes. Three codons in the PRO and two in the RT were located in drug resistance-associated sites that are also located within CD8^+^ T-cell epitopes. Similar results were obtained when the selection analysis was performed in the divided dataset using the SLAC method in Datamonkey; this method identified as positively selected the same positions previously identified by the HyPhy method implemented in MEGA 5.2.2, except for codon 202 of the RT.

### 3.2. Identification of CD8^+^ T-cell Epitopes with Amino Acid Substitutions

Based on the previous analysis, we selected the mutations located within CD8^+^ T-cell epitopes with significant (*p* < 0.05) evidence of positive selection and with a frequency greater than 5% among the 614 sequences. There were 20 such mutations, 11 in the PRO and 9 in the RT (Table 2).

**Table 2:** Identification of epitopes affected by the positively selected mutations in in PRO and RT.

Mutations	Frequency (%)	Epitope Affected (HLA Alleles) ^a^	Association ^b^
**Protease**			
I13V	20.4	QRPLVTIKI (A*01:01)	NC
		QRPLVTIKIG (B51)	NC
		VTIKIGGQLK (A*11:01)	SF
		TIKIGGQLK (A3 supertype)	NC
L19I	9.0	VTIKIGGQLK (A*11:01, A*03:01)	SF
		TIKIGGQLK (A3 supertype)	NC
E35D	28.7	DTVLEEMSL (A*68:02)	NC
		EEMSLPGRW (B*44:02, B*44:03, B18, B40)	IE
S37N	56.0	DTVLEEMSL (A*68:02)	SF
		EEMSLPGRW (B*44:02, B*44:03, B18, B40)	SF
S37D	14.1	DTVLEEMSL (A*68:02)	NC
		EEMSLPGRW (B*44:02, B*44:03, B18, B40)	SF
R41K	42.8	EEMSLPGRW (B*44:02, B*44:03, B18, B40)	NC
		LPGRWKPKMI (Cw3)	NC
I54V^c^	19.3	KMIGGIGGFI (B62)	IE
I72V	9.3	IEICGHKAIG (B18, B40, B44)	NC
		GHKAIGTVL (B15)	NC
I72T	5.5	IEICGHKAIG (B18, B40, B44)	NC
		GHKAIGTVL (B15)	NC
V77I^c^	27.8	LVGPTPVNI (A2)	NC
V82A ^c^	13.8	LVGPTPVNI (A2)	IE
Reverse Transcriptase			
T39A	7.1	ALVEICTEM (A*02, A*02:01, A2)	NC
A98S	8.7	GIPHPAGLK (A*03:01)	NC
V118I	19.1	VLDVGDAYFSV (A*02:01)	NC
		DAYFSVPL (A24, B*51:01)	NC
I135T	30.7	KYTAFTIPSI (A2)	NC
		TAFTIPSI (B*51)	IE
I35V	7.7	KYTAFTIPSI (A2)	NC
		TAFTIPSI (B*51)	IE
S162C	10.1	SPAIFQSSM (B7, B35)	SF
		AIFQSSMTK (A*03:01)	SF
T200A	19.0	DLEIGQHRTK (A3)	NC
I202V	6.8	KIEELRQHL (A2)	NC
		KIEELRQHLL (B58)	NC
		IEELRQHLL (B*40:01, B60, B61)	IE
R211K	49.3	EELRQHLLRW (B44)	NC

^a^ The residues in the epitope affected by the substitutions are in bold. ^b^ IE: Immune escape, *i.e.*, mutations with documented or inferred escape; SF: susceptibility forms, defined as epitope sequences with mutations able to induce a CTL response; NC: none classified, *i.e.*, mutations not included in the previous definitions. ^c^ Mutations associated with both, immune escape and drug resistance.

Some of these mutations were located in more than one overlapping epitope; for instance, the I13V mutation was found in four peptides, although only VTIKIGGQLK had been previously recognized as an epitope binding to different HLA molecules and influencing the immune response [58]. As a consequence 31 epitopes were affected by mutations at a high frequency. In addition, there was more than one mutation in the some epitopes, for example in DTVLEDMNL and EDMNLPGRW.

Only five mutant epitopes were previously recognized as immune escape (IE) and seven as susceptible forms (SF). Most (19) of the mutated epitopes have not been classified as IE or SF. All epitopes identified in this study as positively selected are presented by HLA alleles that have been reported in our population, except the epitope LPGRWKPKMI which is presented by HLA-Cw3; frequencies of alleles at the HLA-C locus have not been reported for Colombia.

### 3.3. Docking Simulation and Algorithmic Estimation of the Affinity of Peptides Binding to HLA Molecules

**Figure 1:**
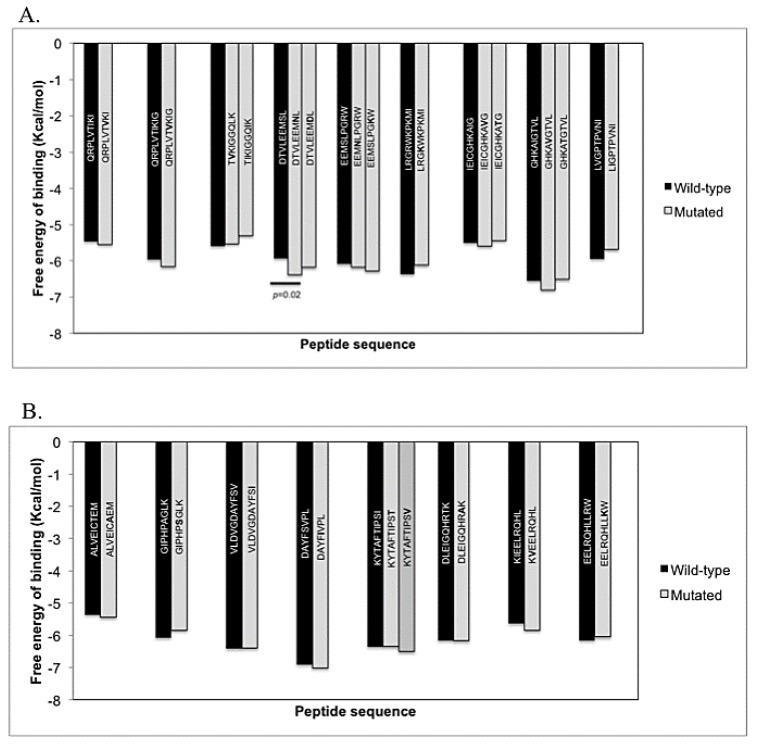
Free energy of binding to HLA molecules of peptides using the *in silico* assay. The mean of the lowest free energy of binding in 20 runs was plotted for each epitope derived from the PRO (**A**); and RT (**B**).

We implemented an *in silico* screening system to assess the affinity between the HLA proteins and the corresponding WT or mutated peptides, using a combination of different computational tools. This protocol was applied to peptides selected in Table 2. First, each peptide with the respective HLA molecule was subjected to docking analysis in AutoDockVina and the average of the data was plotted (Figure 1). All the evaluated peptides, either WT or mutated, were docked to the respective HLA molecule (Figure 1). Average free energy of binding ranged from −4.0 to −7.7 Kcal/mol for the PRO and RT peptides. No significant differences in values of free energy between the WT and the mutated forms of the epitopes where found, except for the DTVLEEMNL variant, which has a decrease of 0.44 Kcal/mol (*p* < 0.05) from the wild type DTVLEEMSL form (Figure 1A,B).

Next, we estimated the binding affinity using three algorithmic tools: SMM, NetMHC and NetMHCpan. Many epitopes had an IC_50_ > 500 nM, indicating a low binding affinity; only one epitope in the PRO and six in the RT had good predicted binding affinities (IC_50_ < 50 nM) by at least one of the algorithms (Table 3). Four mutated epitopes in the PRO and three in the RT exhibited a significant decrease in the predicted affinity with respect to the WT form, as assessed by a two-fold or greater increase in the IC_50_ in at least two algorithms. Four of these epitopes, two in the PRO (TIKIGGQIK, DTVLEEMDL) and two in the RT (VLDVGDAYFSI, KYTAFTIPST**)** had not been reported previously as IE mutations (Table 3).

The IC_50_ estimated by the three algorithmic methods were highly correlated, with *r* ≥ 0.6991 and *p* < 0.0001. Conversely, the correlations of these values with the docking’s ΔGs were all negatively but not significantly correlated (Supplementary Table S1).

**Table 3:** Epitope-HLA binding affinities by three computational algorithms (SMM, NetMHC and NetMHCpan).

Amino Acid Sequence	Alleles	SMM	NetMHC	NetMHCpan
		Affinity (nM)	Affinity (nM)	Affinity (nM)
LPPVVAKEI ^a^	B*51	172	102	797
NLVPMVATV ^a^	A*02	66	29	21
Protease								
QRPLVTIKI	A*01:01	194334	21837	36562
QRPLVTVKI		231499	21639	37194
QRPLVTIKIG	B51	166360	30751	43536
QRPLVTVKIG		168286	30708	43446
TIKIGGQLK	A3	432	582	537
TVKIGGQLK		456	720	856
TIKIGGQIK		1041 ^b^	2126 ^b^	1654 ^b^
DTVLEEMSL	A*68:02	874	2686	1709
DTVLEEMNL		1012	3795	2078
DTVLEEMDL		2278 ^b^	12370 ^b^	6676 ^b^
EEMSLPGRW	B*44:02	30	25	14
EEMNLPGRW		32	28	21
EEMSLPGKW		30	22	14
EDMSLPGRW		431 ^b^	565 ^b^	578 ^b^
IEICGHKAIG	B44	2093	6969	6674
IEICGHKAVG		2122	5792	5601
IEICGHKATG		2103	5060	5162
GHKAIGTVL	B15	13091	14972	15778
GHKAVGTVL		9840	13657	14015
GHKATGTVL		8222	12947	14673
LVGPTPVNI	A2	3027	3829	4005
LIGPTPVNI		1945	2195	1596
LVGPTPANI		3555	3014	3912
KMIGGIGGFI	B62	514	415	769
KMIGGIGGFV		1493 ^b^	937 ^b^	1744 ^b^
Reverse transcriptase								
ALVEICTEM	A2	116	70	41
ALVEICAEM		101	50	32
GIPHPAGLK	A*03:01	290	108	316
GIPHPSGLK		266	99	334
VLDVGDAYFSV	A*02:01	4314	284	9
VLDVGDAYFSI		8788 ^b^	534	33 ^b^
DAYFSVPL	B*51:01	7628	6527	4202
DAYFSIPL		15150	7805	4148
KYTAFTIPSI	A2	1470	5199	9216
KYTAFTIPST		4925 ^b^	15627 ^b^	27884 ^b^
KYTAFTIPSV		381	1509	5743
Reverse transcriptase								
TAFTIPSI	B51	996	2399	1153
TAFTIPST		1128	16898 ^b^	17009 ^b^
TAFTIPSV		1372	4052	2887 ^b^
DLEIGQHRTK	A3	798	8188	15564
DLEIGQHRAK		839	9011	15773
KIEELRQHL	A2	5689	10072	8180
KVEELRQHL		8853	13644	14234
KIEELRQHL	A2	5689	10072	8180
KVEELRQHL		8853	13644	14234
EELRQHLLRW	B44	78	104	29
EELRQHLLKW		78	84	29

^a^ Known epitopes of HIV-1 GAG and cytomegalovirus pp65, respectively, included as positive controls of the analyses performance. ^b^ Two-fold or larger differences between the affinities of the WT and the mutated peptide.

## 4. Discussion

In the present study, we explored immune escape mutations in the PRO and the RT, using HIV-1 sequences from 614 Colombian patients who were genotyped for antiviral sensitivity purposes. We found evidence of positive selection in many codons, associated or not with drug resistance (Table 1). All 17 selected substitutions not previously associated to drug resistance were located in CD8^+^ T-cell epitopes. Of these, 14 have not been previously identified as immune escape in the HIV Molecular Immunology Database of Los Alamos [40], opening the possibility of being considered as newly identified escape mutations, and prompting us to consider alternative explanations.

One possibility is that some of these mutations are indeed associated to drug resistance, but they have not been included in the drug resistance databases [43] due to the lack of strong evidence of this association. For instance, the I13V mutation in the PRO, that had been reported as a minor mutation associated with resistance to tipranavir and ritonavir, was removed from the 2010 update [59]. A related explanation is that these mutations could have compensatory effects that had not been previously detected. Since most HIV drug resistance mutations are naturally deleterious, such compensatory mutations are frequently selected. Similarly, compensatory mutations that restore fitness losses have previously been reported in CD8^+^ T-cell escape mutants [26,60].

An alternative explanation is that the selection test could be detecting significant elevations in the d_N_/d_S_ ratio just for stochastic reasons; in a 304 codon-long sequence with a significance threshold of 0.05 one could expect 15 codons with a d_N_/d_S_ < 0.05 just by chance. However, even with the strict Bonferroni correction for multiple comparisons, most (11/18 in the PRO and 7/17 in the RT) codons remain with a d_N_/d_S_ ratio with a *p*-value below the corrected (0.00016) significance level in the test for positive selection, as shown in Table 1.

A more arguable explanation for this finding is the potential “hitch-hiking” effect of selective sweeps induced by the selective pressure exerted by antiviral drugs. In the presence of antiviral therapy, a drug resistance mutation will increase its frequency in the viral population up to fixation; other mutations arising in the same molecule, could hitch-hike along with it to fixation even if they are slightly deleterious [61]. Previous reports indicate that such hitch-hiking mutations persist for about 18 months in HIV-infected patients receiving antiviral therapy [62]. This effect is attenuated by the high rate of recombination, which is even higher than the mutation rate in terms of events per genome per HIV-1 cycle [63]; this could limit the effect of selective sweeps to closely linked sites. However, the extent of this effect is difficult to assess and we cannot rule out the possibility that this phenomenon has played a role in the mutations observed in our dataset.

Even so, the consistent location of non-synonymous substitutions in CD8^+^ T-cell epitopes, and not in other sites that could also be affected by hitch-hiking, still makes the immune escape hypothesis a plausible explanation for the high frequency of such mutations. Although not all mutations were located at the second position or at the C-terminal amino acid of the peptide chain that are the critical positions for the stability of the HLA-epitope interaction [64], the immune escape could also be associated with lack of T-cell receptor (TCR) recognition. Previous reports indicate that some mutations in the epitope can alter TCR recognition [65,66], resulting in a reduced functional capacity of CD8^+^ T-cells [67,68]. Functional studies are required to explore whether or not these mutations alter the strength of the interaction between the TCR and the HLA-peptide complex.

CD8^+^ T-cells play a crucial role in the control of HIV replication [69], although complete viral suppression is not achieved for a number of different reasons, including the appearance of mutations in immunodominant epitopes [70]. Several studies have demonstrated that HIV-specific CD8^+^ T-cell response selects escape mutants during the acute and chronic phases of HIV and simian immunodeficiency virus (SIV) infections [68,71,72], inducing loss of immune control and disease progression [73]. The selection of escape mutations depends on the HLA molecules. Indeed, the escape mutations could be predicted based on the host MHC alleles [74]. Most escape mutations revert when the virus is transmitted to another host with a different HLA make up. However, for HLA alleles that are common in a population, the corresponding escape mutations may become predominant in the strains circulating in that particular region, contributing to viral evolution and inducing an imprinting effect [25,75].

In this study, most CD8^+^ T-cell epitopes affected by positively selected mutations are restricted to the HLA alleles found in high frequency in the Colombian population (Supplementary Table S2). For instance, several mutated peptides are presented by the HLA-A*02 and -A*03 molecules, which in Colombia have frequencies of 22.2% and 7.9%, respectively [76]. In the case of HLA-B alleles, we observed mutations in epitopes restricted to the molecules -B*18 (7.1%), -B*35 (17.8%), -B*44 (9.1%) and -B*51 (6.4%) that are found in high frequencies (shown in parenthesis) [76]. However, the correlation between the frequency of the HLA alleles and the frequency of the mutation is not perfect. Only one HLA-A*24-restricted and one HLA-A*68-restricted epitopes showed substitutions in our study; these alleles had frequencies of 19.1% and 5.6%, respectively in Colombia; this could be due to a higher functional constraint against mutations in these peptides. Moreover, some epitopes restricted to rare HLA alleles in our population, such as HLA-B*15 (0.6%), were mutated with a significant (5.5%) frequency; these mutants could have appeared in a viral lineage before its introduction to Colombia and its frequency could have persisted or even increased by random genetic drift.

To predict the possible functional consequences of the observed amino acid substitutions in CD8^+^ T-cell epitopes, and further substantiate their immune escape potential, we performed an *in silico* docking simulation assay between the HLA molecules and the WT and mutated peptides. In addition, we predicted changes in the affinities of binding using three computational algorithms. We observed discrepancies between the inferred affinities obtained by docking simulation and by the computational algorithms; no correlation was observed in the values obtained by these two strategies. While the docking showed almost no changes in the free energy of binding in the mutant peptides, the algorithms predicted a significant decrease in affinities in seven peptides, four of which (TIKIGGQIK, DTVLEEMDL, VLDVGDAYFSI and KYTAFTIPST) had not been previously reported as immune escape mutants. Moreover, the results of the three algorithmic methods were strongly correlated (Supplementary Table S1).

The discrepancies between strategies could be explained by their different approaches. In general, the algorithms generate a prediction from a set of experimental data [77] derived from previous reports in-house experiments or specialized databases containing epitope-related information; estimates are expressed as a function of the concentration of a binding inhibitor [78]. The docking strategy is based on computational structural analysis; it predicts the affinity based on the spatial disposition of the atoms and chemical bonding, treating the protein as a rigid structure and estimating the free energy of binding; the accuracy of this kind of prediction could be affected by the size of the ligand, the dimension of the motion space or the rotational degrees of freedom of the ligand [79].

In this study, the algorithmic methods detected some (three out of five) of the previously reported IE mutations and allowed to infer four other mutations not previously reported as such. The docking strategy did not identify any of these IE mutations; instead, it detected a modest change of 0.44 kcal/mol between the WT and mutated forms in an epitope catalogued as SF. This suggests that algorithmic methods are more sensitive as tools for predicting potential IE mutants; however, we observed that some epitopes that experimentally had high binding to HLA molecules and high immunogenic capacity, exhibited low predicted affinities by the computational algorithms. For example, LVGPTPVNI was previously reported with a high HLA-A2 binding affinity and cytotoxic response of CD8^+^ T cells [80]; however, all the predicted affinities had IC_50_s > 500 nM (SMM: 3027.68 nM, NetMHC: 3829.00 nM and NetMHCpan: 4005.25 nM). Taking this into account, the four mutations found here with both, strong evidence of positive selection and significant decrease in the affinity of the epitopes, should be regarded only as candidate IE mutations; *in vitro* assays are required to determine whether they lead to destabilization of the HLA-peptide-TCR complex or to changes in the functional profiles in CD8^+^ T-cells.

The main limitations of this study were the absence of HLA typing, CD4^+^ T-cell counts and clinical data of the patients from whom the HIV-1 sequences were obtained, which would have allowed to make associations between mutations and disease progression. Nevertheless we can assume that all patients had viral loads ≥ 1000 RNA copies/µL, the minimal viral load that permits sequencing with the TRUGENE^®^ kit. On the other hand, the strength of the bioinformatic approach to predict immune escape mutants that we introduce here is the combination of several analytical tools of different nature, and its application to a large dataset.

In summary, we introduced a strategy aimed at screening immune escape mutations that combine methods for positive selection, protein-protein docking and computational algorithms for affinity prediction. This allowed us to postulate four possible new mutations associated with immune escape in HIV-1-infected patients. Functional studies should be performed to confirm the effect of these mutations on the response of CD8^+^ T-cells; prospective clinical studies could test the biological relevance of these findings.

## Supplementary Files

Supplementary File 1
