# Supplementary material for: Selection Pressure in CD8+ T-cell Epitopes in the pol Gene of HIV-1 Infected Individuals in Colombia. A Bioinformatic Approach
Source: Viruses. 2015 Mar 20;7(3):1313–31. doi: 10.3390/v7031313 (PMC4379572; doi:10.3390/v7031313)
Supplement: Supplementary File 1 [file viruses-07-01313-s001.pdf]

Supplementary Files

Table S1. Correlation between epitope-HLA predicted affinities.

Analysis	Correlation Coefficient (r)	*p* Value
NetMHCpan *vs.* Docking	−0.1264	0.4494
SMM *vs.* Docking	−0.1004	0.5487
ANN *vs.* Docking	−0.1094	0.5134
NetMHCpan *vs.* SMM	0.6991	<0.0001 *
NetMHCpan *vs.* ANN	0.9315	<0.0001 *
SMM *vs.* ANN	0.8615	<0.0001 *

* $p < 0.05$.

Table S2. Allele frequency distribution of the HLA-A and -B in Colombia [1].

Alleles	Frequency (%)
HLA-A	
01	5.1
02	22.2
03	7.9
10	0.1
11	5.0
23	7.2
24	19.1
25	1.1
26	3.3
29	3.7
30	6.7
31	4.2
32	1.8
33	4.1
34	0.6
36	0.4
43	0.1
66	0.6
68	5.6
69	0.4
74	0.9
80	0.1

Table S2. *Cont.*

Alleles	Frequency (%)
HLA-B	
07	4.8
08	3.0
13	1.0
14	0.3
15	0.6
17	1.1
18	7.1
27	1.4
35	17.8
37	0.3
38	2.4
39	4.9
40	1.1
41	1.4
42	0.8
44	9.1
45	1.1
46	0.1
47	0.2
48	0.6
49	2.9
50	1.5
51	6.4
52	1.1
53	2.1
55	0.9
56	0.3
57	2.5
58	2.0
59	0.2
60	3.4
61	5.4
62	1.7
63	1.7
64	1.1
65	5.2
67	0.1
70	0.4
71	0.4
72	1.4
75	0.1
78	0.2
81	0.1

Reference

1. Rodriguez, L.M.; Giraldo, M.C.; Garcia, N.; Velasquez, L.; Paris, S.C.; Alvarez, C.M.; Garcia, L.F. Human leucocyte antigen gene (HLA-A, HLA-B, HLA-DRB1) frequencies in deceased organ donors. *Biomedica* **2007**, *27*, 537–547.
